# BRL3 and AtRGS1 cooperate to fine tune growth inhibition and ROS activation

**DOI:** 10.1371/journal.pone.0177400

**Published:** 2017-05-18

**Authors:** Meral Tunc-Ozdemir, Alan M. Jones

**Affiliations:** 1 Department of Biology, University of North Carolina at Chapel Hill, Chapel Hill, NC, United States of America; 2 Department of Pharmacology, University of North Carolina at Chapel Hill, Chapel Hill, NC, United States of America; Iowa State University, UNITED STATES

## Abstract

Plasma membrane-localized leucine-rich repeat receptor-like kinases directly activates G protein complex via interaction with seven transmembrane domain Regulator of G-protein Signaling 1 (AtRGS1) protein. Brassinosteroid insensitive 1 (BRI1) LIKE3 (BRL3) phosphorylates AtRGS1 *in vitro*. FRET analysis showed that BRL3 and AtRGS1 interaction dynamics change in response to glucose and flg22. Both BRL3 and AtRGS1 function in glucose sensing and *brl3* and *rgs1*-2 single mutants are hyposensitive to high glucose as well as the *brl3/rgs1* double mutant. BRL3 and AtRGS1 function in the same pathway linked to high glucose sensing. Hypocotyl elongation, another sugar-mediated pathway, is also implicated to be partially mediated by BRL3 and AtRGS1 because *rgs1*-2, *brl3*-2 and b*rl3*-2/ *rgs1*-2 mutants share the long hypocotyl phenotype. BRL3 and AtRGS1 modulate the flg22-induced ROS burst and block one or more components positively regulating ROS production because the *brl3/rgs1* double mutant has ~60% more ROS production than wild type while *rgs1*-2 has a partial ROS burst impairment and *brl3* has slightly more ROS production. Here, we proposed a simple model where both BRL3 and AtRGS1 are part of a fine-tuning mechanism sensing glucose and flg22 to prevent excess ROS burst and control growth inhibition.

## Introduction

The Arabidopsis genome encodes more than 200 leucine-rich repeat receptor-like kinases (LRR RLK) and many are known to regulate developmental and defense-related processes including cell proliferation, stem cell maintenance, hormone perception, host-specific as well as non-host-specific defense responses, wounding response, and symbiosis [1]. Considerable indirect evidence links RLKs to G protein signaling pathways which prompted us to propose a previously-unknown mechanism. Specifically, we proposed direct activation of the G protein complex through a seven-transmembrane domain Regulator of G-protein Signaling 1 (AtRGS1) protein phosphorylation by RLKs in what we termed the “mix and match” model for signal discrimination [2]. A screen of 70 active, recombinant arginine-aspartate type LRR RLKs discovered several RLKs that phosphorylate AtRGS1 *in vitro* and one of them is is related to brassinosteroid insensitive1 (BRI1) and is designated BRI1-LIKE3 (BRL3) [3]. BRL3 is one of four members of the plant hormone brassinosteroid (BR) receptor family in Arabidopsis [4–7].

Although BRL3 phosphorylates AtRGS1 *in vitro*, the consequences of this interaction are not known. Heterotrimeric G proteins control growth, cell proliferation, abiotic and biotic stress and hormone responses and glucose sensing [8]. Brassinosteroid (BR) and glucose regulate various common responses in plants [9]. Therefore, we hypothesized that BRL3 and AtRGS1 may function in BR and glucose crosstalk. However, *rgs1* mutants have wild type sensitivity towards BR [9]. Therefore, we investigated a possible role for BRL3 in AtRGS1-mediated glucose sensing independent of BR. The G protein pathway represents one of the mechanisms in plants to detect and respond to changes in sugar dose and duration [10–12]. AtRGS1 internalizes in response to D-glucose upon phosphorylation by AtWNK8, which is one of 11 WNK (WITH NO LYSINE) family Ser/Thr kinases in Arabidopsis [13]. *rgs1*-2 mutant plants are hyposensitive to D-glucose [14] whereas *gpa1*-3 is hypersensitive to D-glucose [15]. G protein signaling is also directly activated by BAK1, the binding partner to BRL3 [5], in response to flg22, a 22-amino acid bacterial flagellin peptide [3]. Therefore, we also considered that BRL3 and AtRGS1 function together in flg22 responses.

Loss of function alleles for BRL3 and AtRGS1 do not have gross developmental phenotypes [4,16] and therefore due to a lack of pleotrophy, they are useful for studies on signaling. BRL3 binds brasinolide but has otherwise not yet been implicated in a signaling pathway other than for brassinosteroid.

Here, we report high glucose and flg22 responses of *brl3* mutants and the *brl3*/*rgs1* double mutant and dynamics of BRL3/AtRGS1 complex in response to these ligands. *brl3* and *brl3*/*rgs1* mutants were hyposensitive to high glucose similar to *rgs1*-2 mutants. These results suggest a direct role for G-protein/BRL3 complex in sugar signaling. The flg22-induced ROS burst is slightly higher in *brl3* mutants and vice versa in the *rgs1* mutants. On the other hand, the *brl3*/*rgs1* double mutant produces ~60% more ROS in response to flg22. This suggests that in the absence of BRL3 and AtRGS1, there is release of inhibition of an unknown component that positively regulates flg22 induced ROS burst. In brief, BRL3 and AtRGS1 work together to fine tune growth inhibition and ROS production.

## Materials and methods

### Plant materials and growth conditions

*Arabidopsis thaliana* (Arabidopsis) Col-0 and T-DNA insertion null mutants *rgs1-2* [17] and *brl3-1 and brl3-2* (*SALK_079612C* and *SALK_006024C* (with no full length coding sequence, transcript or genomic DNA as shown on S1A and S1B Fig) respectively), *brl1 (SALK_005982)*, *fls2 (SAIL_691_C4)* [18], *bak1*-4 (SALK_116202) [19], and *bak1*-5 [20]. No full-length coding sequence, transcript, or genomic DNA was detected in *brl3-2* as shown on S1A and S1B Fig. The AtRGS1 open reading frame (At3g26090) behind the CaMV 35S promoter with a C-terminal YFP tag in pEG101 Gateway®-compatible destination vector was overexpressed in *Arabidopsis thaliana* (Arabidopsis) Col-0 wild type [13] or *brl3-2* plants and labeled wt*/OE* and *brl3-2/ OE*, respectively. Where indicated, the media contained 0% or 6% glucose or mannitol and seedlings were grown vertically under continuous dim-light (20–40 μEinstein/m^2^/s). For root growth analysis every 24 h, the root tips were marked until the 5^th^ day. The root lengths were calculated using ImageJ software. The same plants on their 8^th^ day were assessed for the “green seedling” assay [14]. *Nicotiana benthamiana* plants used for the BiFC and FRET experiments were grown at 26°C under fluorescent light 16 h light (120 μEinstein/m^2^/s) and 8 h dark.

### Imaging

Transient expression in *N*. *benthamiana* for Förster Resonance Energy Transfer (FRET) was performed as previously described [12,21]. Briefly, *Agrobacterium* carrying a binary plasmid encoding either AtRGS1-YFP, BRL3-CFP, or P19 (viral RNA silencing suppressor (Shamloul et al., 2014)) were infiltrated in 10 mM MgCl_2_, 10 mM 2-(N-morpholino) ethanesulfonic acid and 200μM acetosyringone buffer into the abaxial sides of 4–5 week-old *N*. *benthamiana* leaves with a needleless 1-mL syringe. The mitochondrial RFP marker Mt-rk, obtained from the Arabidopsis Biological Resource Center (CD3-991), was used as an internal positive transformation control for BiFC along with nYFP- and cYFP-tagged proteins. On the 4th to 6th day post-agro infiltration, confocal imaging of leaf discs incubated in 1 μM flg22 or different doses of glucose for various time points was performed using a Zeiss LSM710 confocal laser scanning microscope equipped with a C-Apochromat 40X/1.20NA water immersion objective. A 489-nm and a 561-nm diode laser was tuned to excite YFP and mcherry, respectively. Emission was detected at 526–563 nm (YFP) and 583–622 nm (mcherry) by a photomultiplier tube detector. For acceptor photobleaching, a 514-nm and a 458-nm argon lasers was tuned to excite YFP (acceptor) and CFP (donor), respectively. Emissions were detected within the range of 516–596 (YFP) and 460–517 (CFP) nm. Region of interests were photobleached by scanning for 100 iterations with a 514-nm argon laser line at 100% power with a pinhole diameter set to 1.00 airy units. Acceptor photobleaching decreased YFP channel intensity to ~20–30% of its initial value. FRET efficiency % was then calculated via Zen Software (http://www.zeiss.com/microscopy/en_de/downloads/zen.html).

### ROS burst assay

The flg22-induced ROS burst was measured according to Chung and coworkers [22] using 16–24 leaf discs from 6- to 8-week-old Col-0, *fls2*, and *brl3* mutant plants. Reaction mix (100 μl of 17 μg/ml of luminol [Sigma; A8511], 10 μg/ml of horseradish peroxidase [Sigma; P6782], and 1 μM flg22 (ChinaPeptides) was added to leaf discs incubated with water overnight in a 96-well plate. Luminescence was measured immediately with 1 s integration at 2 min intervals over 48 min using a SpectraMax luminometer (Molecular Devices).

## Results

### BRL3 interacts with AtRGS1 *in vivo*

**Fig 1:**
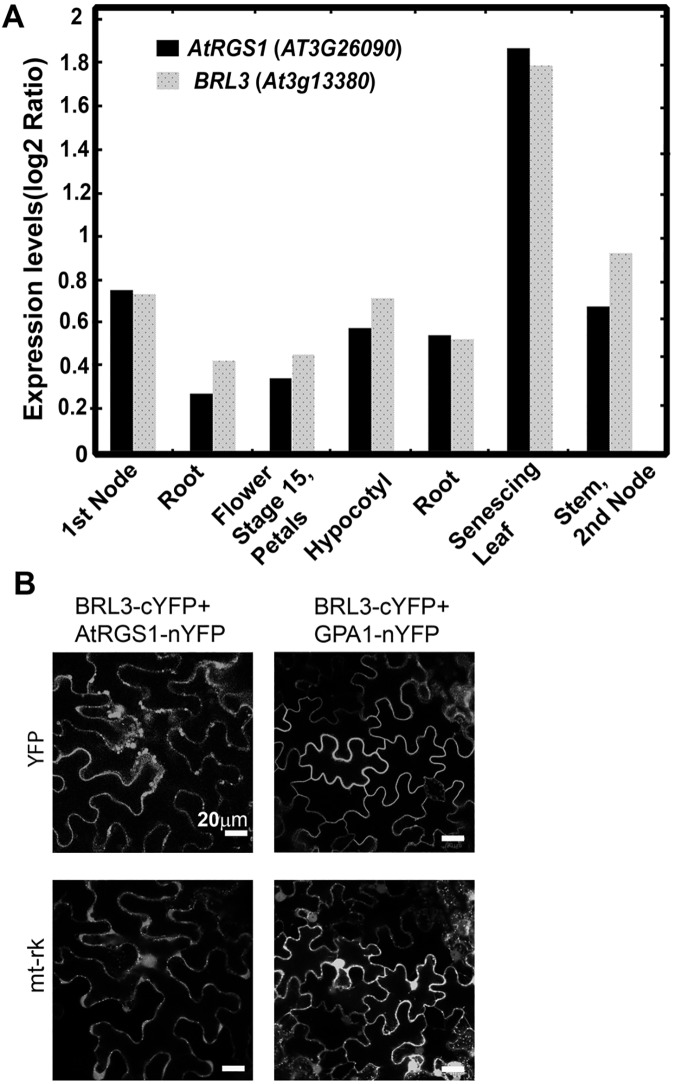
BRL3 interacts with AtRGS1 *in vivo*. **(A)** BRL3 and AtRGS1 are co-expressed in several tissues. Arabidopsis eFP Browser [38] available at http://www.bar.utoronto.ca/ was used for exploring Arabidopsis microarray data to visualize *BRL3* and *AtGRS1* gene expression profile.(**B)** BRL3 complements fluorescence by AtRGS1 and GPA1 in BiFC. Confocal images of *N*. *benthamiana* cells expressing BRL3-cYFP and AtRGS1-nYFP or BRL3-cYFP and GPA1-nYFP pairs. The upper row indicates the YFP complementation results and the lower row is a transformation reporter, mitochondria protein tagged with mCherry (mt-rk).

Because BRL3 phosphorylates AtRGS1 [3] and potentially regulates G protein signaling and because *AtRGS1* and *BRL3* are both expressed in the 1^st^ node, root, hypocotyl, flower stage 15/ petals, stem/2^nd^ node, and senescing leaf (Fig 1A), we investigated its functional interaction with AtRGS1. First, we checked if AtRGS1 physically interacts with BRL3 *in vivo* by bimolecular fluorescence complementation (BiFC). BRL3 interacted with AtRGS1 *in vivo* (Fig 1B) whereas a negative control membrane protein, AtHIR2 did not (S2 Fig). AtRGS1-nYFP showed no signal when it was expressed alone in *N*. *benthamiana*. The tagged Gα subunit, GPA1-nYFP, interacted with BRL3-cYFP (Fig 1B) while GPA1-nYFP by itself did not complement flouresence (S2 Fig).

### BRL3 and AtRGS1 modulate glucose induced growth inhibition

**Fig 2:**
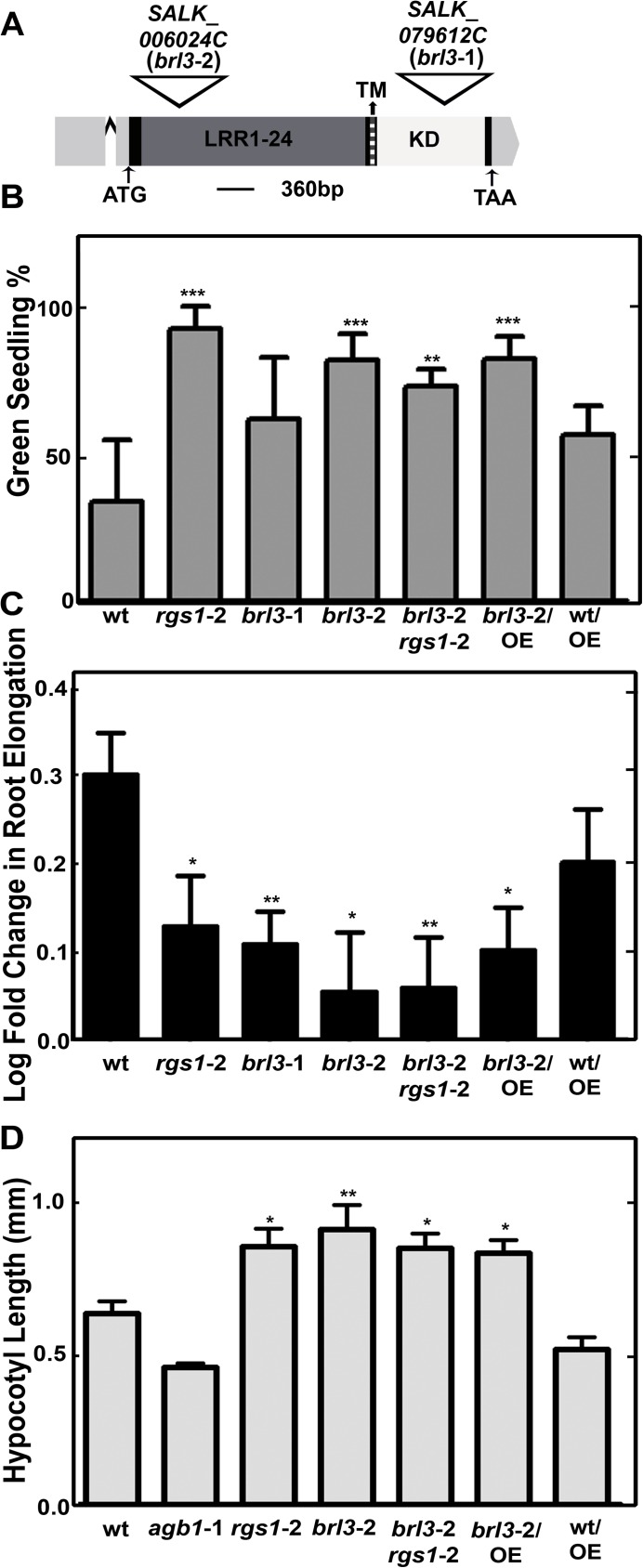
BRL3 and AtRGS1 controls glucose responses. **(A)** T-DNA insertion position in *brl3*-1 and *brl3*-2 on the 4367-bp-gene model of BRL3 (http://www.arabidopsis.org/). Regions encoding 24 predicted leucine-rich repeats (LRR), transmembrane (TM, dark gray region) and kinase domains (KD, indicated in white) are labeled accordingly. The 5’ and 3’ UTRs are highlighted in light gray and the single intron is indicated with a carat (^). Both T-DNA insertions are in the single exon. (**B)** BRL3 is required for glucose perception. % of green cotyledons of 8-day-old Arabidopsis seedlings grown on ¼ MS media with 6% glucose under continuous dim-light (20–40 μEinstein/m2/s). The averages are from 4 biological replicates with 8 to 30 seedlings for each genotype. Error bars represent standard deviations. One-way ANOVA followed by Tukey tests were used to compare each genotype to wildtype (wt) (**, *** *P* value < 0.001, 0.0001) (**C)** BRL3 and AtRGS1 regulate root growth elengation response induced by high glucose. Log-transformed fold change in root growth of Arabidopsis seedlings grown on ¼ MS media with 6% glucose at continuous dim light between the 4^th^ and 5^th^ day. This experiment (n = 30 to 100) was repeated three times with similar results. Images were captured and ImageJ was used to quantitate lengths OE means overexpression of AtRGS1 in the indicated wild type (Col-0) or *brl3*-2 mutant. Student’s *t*-test was used to compare each genotype to wt (*, ** *P* value < 0.05, 0.01). **(D)** Seedlings were grown on ½ × MS plates with 1% sucrose for 2 d in darkness. Length of hypocotyls was measured and quantified by image J. Data are mean ± SEM. One-way ANOVA followed by Tukey tests were used to compare each genotype to wt (*, ** *P* value < 0.05, 0.001).

The loss-of-function alleles of *AtRGS1* confer glucose hyposensitivity while all G protein subunit mutants are hypersensitive to glucose inhibition of early seedling development and root elongation [17]. If BRL3 is also involved in glucose sensing, we expect that loss of BRL3 will confer a differential glucose response in plants. We isolated two new T-DNA insertion mutants (Fig 2A), *brl3*-1 (SALK_079612C; highly reduced expression) and *brl3*-2 (SALK_006024C; no full length transcript detected) (Fig 2A and S1A Fig) and tested for high glucose responses (Fig 2B and 2C). First, we used the standardized green seedling assay described by Moore and coworkers [23] to understand the role of BRL3 in glucose signaling. The average percentage of seedlings growing on 6% glucose and showing green cotyledons was determined. Both *rgs1*-2 and *brl3* mutants were less responsive to 6% glucose than wild type seedlings (*P* value < 0.0011) as shown in Fig 2B.

In the absence of glucose the single *brl3* mutants and *brl3/rgs1* double mutant had shorter roots (S3A and S3B Fig) suggesting a positive regulatory function for BRL3 and AtRGS1 in root growth. In *bri1 brl1 brl3* mutants, reduced cell expansion was suggested to be responsible for the overall short root phenotype of BR signaling mutants [7]. The *brl3/rgs1* short root phenotype is consistent with this report of impaired cell expansion in the *bri1 brl1 brl3* triple mutant. However in the presence of glucose, the *brl3* roots were not shorter than wild type (S2D Fig). Glucose reduced elongation of 5-day-old wild type root by 2 fold whereas it decreased elongation by ~1.4 fold in *rgs1*-2, *brl3*-1, *brl3*-2 and *brl3*-2/ *rgs1*-2 roots (*P* value < 0.05 to 0.001) (Fig 2C). Overexpression of AtRGS1 did not compensate for the loss of BRL3 function in either the green seedling assay or the root growth assay. A nontoxic nonmetabolized sugar, mannitol, was used as an osmotic control [24]. The mannitol effect on root growth was slightly more pronounced than that of glucose (~3.3 fold reduction; 0.5 in log scale). Consistent with a previous report on *brl3* mutants and mannitol [25], the osmotic control mannitol decreased the root elongation equally for all genotypes tested (S3C Fig).

We tested early hypocotyl growth of the *rgs1*-2, *agb1*-2 (Gβ subunit), *brl3*-2 single and *brl3*-2/*rgs1*-2 double mutants because this development is partially mediated by G protein-mediated sugar signaling [9,26,27]. Hypocotyl length of seedlings grown on ½ × MS plates with 1% sucrose for 2 d in darkness was measured (Fig 2D). Both *rgs1*-2 and *brl3*-2 mutants had longer hypoctyls (*P* value < 0.05 to 0.001) and the similar hypocotyl phenotype of *brl3*-2/ *rgs1*-2 double mutants to single mutants suggested that BRL3 and AtRGS1 function in the same pathway regulating hypocotyl growth. Because AtRGS1 is phosphorylated by BRL3 [3] and overexpression of AtRGS1 in *brl3*-2 transgenic lines conferred no effect on the hypocotyl phenotype of *brl3*-2, we speculated that BRL3 regulates hypocotyl growth via AtRGS1.

### BRL3 and AtRGS1 affect flg22 induced ROS burst

**Fig 3:**
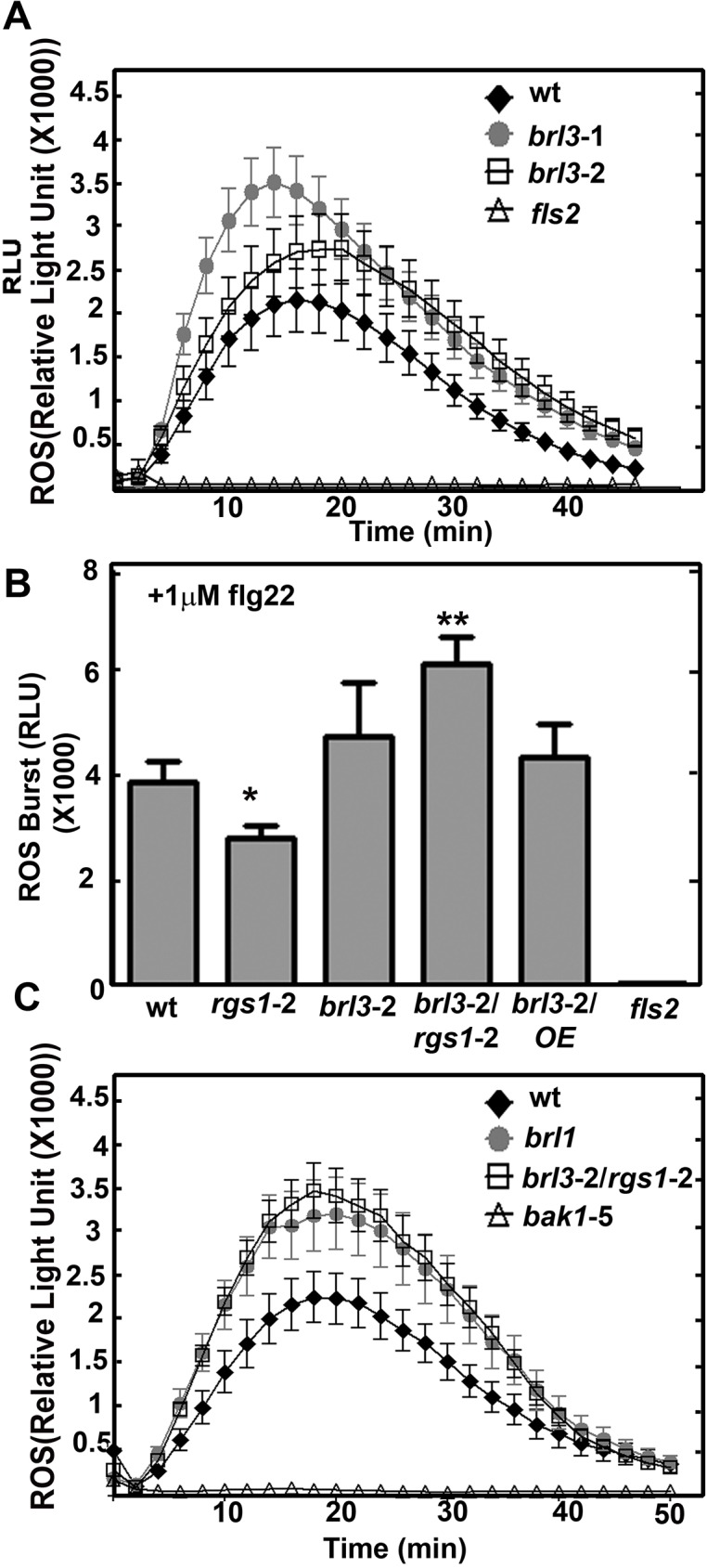
flg22-induced ROS burst is regulated by BRL3 and AtRGS1. **(A)** ROS burst in response to 1 μM flg22 in leaf discs of *brl3* mutants are slightly higher than wild type (n = 16 to 24). 1 μM flg22 triggers a rapid (within 2–4 minutes) and transient oxidative burst in leaf discs obtained from 6- to 8-week-old Col-0 (wt) but not in *fls2* mutants, where the receptor for flg22, FLS2, is genetically ablated. The ROS burst in *brl3* mutant plants were elevated slightly but not significantly. (**B)** ROS burst is fine tuned by BRL3 and AtRGS1. Max ROS burst peak (18^th^ min) in response to 1 μM flg22 in leaf discs of *brl3*-2/rgs1-2 mutants are significantly higher than wild type (n = 16 to 24). (**C)** ROS burst is negatively regulated by another member of the BRI1 family, BRL1. *brl1* mutants show significantly higher ROS burst than wild type (n = 16 to 24). The point mutation in flg22 co-receptor, BAK1, leads to dimished ROS burst in *bak1*-5 mutants thus these plants serve as negative control for ROS assay. Measurement of the ROS burst was carried out using the luminol-based luminescence protocol described in Materials and Methods.

Because plants with an *rgs1* null mutation showed a slight decrease in flg22-induced ROS production [28], we quantitated this flg22-induced ROS response in the *brl3* mutants. The peak of ROS production at 1μM flg22 induction was slightly but not significantly increased in *brl3* leaf disks (Fig 3A). This slight increase was reproducibly observed in three replicate experiments. Peak ROS production quantitated at the 18^th^ minute in response to 1μM flg22 was plotted for wild type plants, *brl3*-2 and *rgs1*-2 single and *brl3*-2*/rgs1*-2 double mutants (Fig 3B). The S3D Fig provides the data for the entire time course. There was ~ 30% less ROS production in *rgs1*-2 (*P* value < 0.05) while ~23% more ROS was produced in *brl3*-2 mutants compared to wild type plants. Remarkably *brl3*-2*/rgs1*-2 had ~ 60% more ROS produced than wild type (*P* value < 0.001). Overexpressing AtRGS1 in *brl3*-2 did not change the higher ROS phenotype of *brl3*-2 and did not have a major affect on this mutant (data not shown). Both AtRGS1 or BRL3 supress a positive ROS burst regulator. Their absence relieves this suppression and leads to ~ 60% more ROS production. We also tested another brassinosteroid insensitive1 (BRI1) homolog, BRI1-LIKE1 (BRL1), which also phosphorylates AtRGS1 *in vitro* [3] to explore its role in the ROS burst. Similar to what we observed with *brl3* mutants, *brl1* also has a large ROS burst in response to flg22 (Fig 3C). This suggests that BRL1 and BRL3 may act together to inhibit ROS production.

### BRL3 and AtRGS1 interaction dynamics change with glucose and flg22

BRL3 phosphorylates AtRGS1 *in vitro* [3] and it interacts with AtRGS1 in BiFC assays (Fig 1B), however the dynamics of this interaction are unknown. Because BRL3 is involved in glucose and flg22 responses, we tested the physical movements of the BRL3/AtRGS1 complex over time after ligand application.

**Fig 4:**
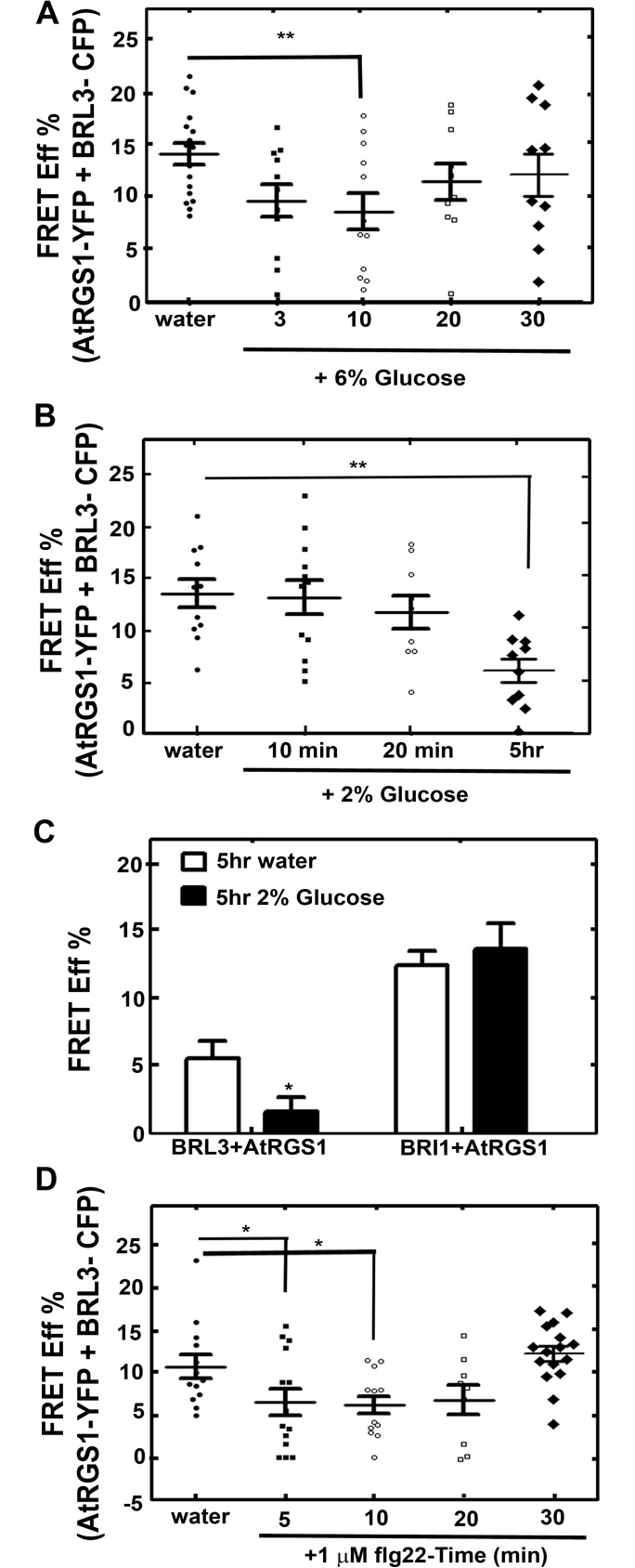
Glucose and flg22 cause conformational change between AtRGS1 and BRL3. **(A)** Glucose (6%) increases the physical distance or orientation between BRL3 and AtRGS1 within 3 to 10 min. FRET analysis was carried out using the acceptor bleaching method in leaf epidermis cells of *N*. *benthamiana* expressing *AtRGS1-YFP* and *BRL3-CFP* transiently as described in Material and Methods. (**B)** 2% Glucose for 5 hr also increases the physical distance or orientation between BRL3 and AtRGS1. **(C)** 2% Glucose does not change FRET Efficiency % between another brassinosteroid receptor kinase, BRI1, and AtRGS1. **(D)** FRET analysis using the acceptor bleaching method in leaf epidermis cells of *N*. *benthamiana* expressing AtRGS1-YFP and BRL3-CFP transiently and treated with 1 μM flg22.

AtRGS1 changes its interaction with WNK kinases in a glucose dose- and time-dependent manner [12]. In brief, high concentrations of D-glucose (6% for 30 min) rapidly signal through AtWNK8 and AtWNK10 phosphorylation of AtRGS1, whereas low, sustained sugar concentration (2% for 5 hr) slowly activates the pathway through phosphorylation of AtRGS1 by AtWNK1, allowing the cells to respond similarly to transient, high-intensity signals and sustained, low-intensity signals. Because AtRGS1 is set in motion by glucose, the interaction dynamics between AtRGS1-YFP and BRL3-CFP in response to high and low levels of sugar over time were quantitated through FRET analyses (Fig 4). Glucose (6%) rapidly (10 min) decreased (*P* value<0.0073) FRET Efficiency (Fig 4A) and this effect took much longer at a lower glucose dose (*P* value<0.0034) (Fig 4B) indicating a change in distance or orientation between the two fluorophores in response to glucose. As a negative control, AtRGS1-YFP and BRI1-CFP (another brassinosteroid hormone receptor) did not respond to glucose (5 hr 2%, Fig 4C) indicating the specificity of BRL3.

AtRGS1 and AtGPA1 interaction is affected by flg22 within 5 min [3]. Therefore, we analyzed the dynamics of the AtRGS1-YFP and BRL3-CFP interaction in response to flg22 over time. AtRGS1-YFP and BRL3-CFP showed a FRET efficiency decrease within the first 5 minute of 1 μM flg22 (*P* value <0.05) (Fig 4D) indicating a change in distance or orientation between these two proteins.

## Discussion

**Fig 5:**
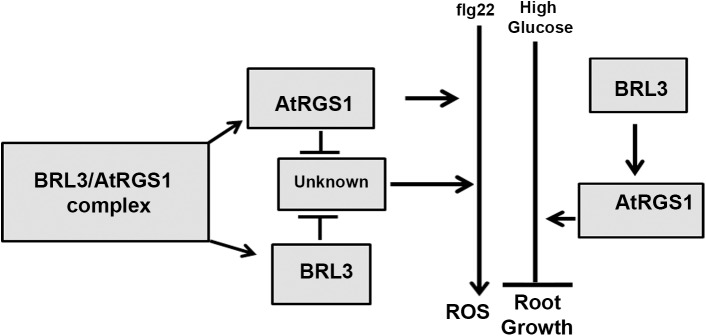
Proposed model for BRL3 and AtRGS1 functions in glucose and flg22 reponses. Partially reduced ROS burst in *rgs1*-2 (*P* value < 0.05) and slightly increased in *brl3*-2 indicate that AtRGS1 is a positive regulator of ROS burst whereas BRL3 is a negative regulator. 60% more ROS than wild type (*P* value < 0.001) is produced in *brl3*-2*/rgs1*-2 (Fig 3B). Absence of AtRGS1 and BRL3 removes the inhibitory effect on an unknown positive regulator of ROS. Removal of BRL3 and AtRGS1 inhibition on this unknown component does not only activates it but also generates a cumulative event by triggering other pathway(s) positively regulating ROS burst.Glucose induced inhibitory effect on root elongation is impaired in plants without AtRGS1 or BRL3. Diminished glucose effect on the single *rgs1*-2 and *brl3*-2 mutants is similar to what is observed with *brl3*-2*/rgs1*-2 double mutants (Fig 2C). This proves that AtRGS1 and BRL3 function in the same glucose response pathway. Because AtRGS1 overexpression in *brl3*-2 mutants results in same phenotype as *brl3*-2, it is concluded that BRL3 acts upstream of AtRGS1.

While it is established that AtRGS1 modulates flg22-induced ROS production [29] and that brasinosteroids inhibit flg22-triggered immune signaling [30], the study here provides genetic evidence for the interaction of BRL3 and AtRGS1 and *in vivo* evidence on the dynamics of this RLK/G protein complex in response to glucose and flg22. As shown in Fig 5, the genetic interactions revealed here provide a complex relationship between BRL3 and AtRGS1 in repression of the ROS burst and growth. Both of the proteins contribute to glucose sensing and root growth. Because loss of either AtRGS1 and BRL3 does not completely abrogate glucose or flg22 responses, these membrane proteins are not absolutely essential for these responses. Rather, we conclude that the function of BRL3 and AtRGS1 is to fine tune the main pathway. The *brl3* and *rgs1*-2 single and the *brl3/rgs1* double mutants are hyposensitive to high glucose (Fig 2B and 2C). We speculate on what else could contribute to glucose- induced fine-tuning pathway mediated by AtRGS1 and BRL3. Arabidopsis EXORDIUM-LIKE1, a BR-regulated gene that is involved in the carbon starvation response [31] and an AtRGS1 interactor as well [32], may also be involved. Given that EXO overexpression increased vegetative growth in comparison to wild-type plants [33] similar to *rgs1*-2 mutants [14,34] and it is required for adaptation to carbon- and energy-limiting growth conditions, it is worthy of investigation of a possible role in the model (Fig 5) in the future. Genetic analysis of the *brl1 brl3 bak1-3* triple mutants showed that BAK1, BRL1, and BRL3 modulate root growth and development [5]. All of these RLKs phosphorylate AtRGS1 *in vitro* [3]. This phosphorylation event may activate G protein subunits that are involved in modulation of cell proliferation, root growth and architecture [35]. Because BRL3 interacts with AtGPA1 *in vivo* (Fig 1A), the functional relevance of this interaction in context related to cell proliferation, root growth and architecture should be fully investigated.

Although AtRGS1 and BRL3 also have independent minor functions in the ROS burst as indicated by the phenotypes of the single mutants, they share the same type of inhibitory function on the unknown positive regulator of ROS burst component (Fig 5). The maximum ROS burst in *rgs1*-2 is slightly reduced (*P* value < 0.05) whereas it is slightly increased in *brl3*-2. Therefore, AtRGS1 is a positive regulator of the ROS burst whereas BRL3 is a negative regulator. Because brassinosteroids inhibit pathogen-associated molecular pattern-triggered immune signaling including the oxidative burst and defense gene expression [30], a slight increase in the ROS burst in the *brl3*-2 mutant is expected. If AtRGS1 and BRL3 are functioning independently, *brl3*-2*/rgs1*-2 is expected to show wild type response. However 60% more ROS than wild type (*P* value < 0.001) is produced in the absence of BRL3 and AtRGS1 (Fig 3C). This indicates that an unknown positive regulator of ROS is freed in *brl3*-2*/rgs1*-2. Thus, we conclude an inhibitory function for BRL3 and AtRGS1 on this unknown component in the flg22 induced ROS burst (Fig 5). This released component starts a domino effect on ROS and triggers other pathway(s) that also positively regulate ROS. Therefore analyzing BRL3 and AtRGS1 function in ROS is just looking at the tip of an iceberg. There are other components waiting to be discovered in ROS burst regulation. To explain the complex genetic interaction between AtRGS1 and BRL3 in the flg22 response, we speculate that both proteins dissociate to interact with the unknown component given that the FRET Efficiency % between this pair drops in response to flg22 (Fig 4D). If so, what are the nature of these components? This unknown would be an interacting partner to both BRL3 and AtRGS1 and a positive regulator of ROS burst. Because the time frame AtRGS1 and BRL3 dissociates is within the period it associates with WNKs and BAK1 in response to glucose and flg22 respectively [12,29], this unknown partner could be WNKs. So it is noteworthy to investigate the links between BRL3 and WNKs and also the competition between AtRGS1 and BRL3 for BAK1 interaction. Given that BRL1 functions similar to BRL3 in the ROS burst (Fig 3C), phosphorylates AtRGS1 and interacts with BAK1 [5], exploring role of BRL1 along with BRL3 is also essential.

Because BRL3 binds BL with high affinity [4] and is redundant to BRL1 and BRI1 in brassinosteroid perception [5], we investigated if AtRGS1 functions along with BRL3 in this pathway. Dose–response curve of exogenous BL treatments (0, 0.1, and 1 nM) of 6-d-old *rgs1-2* seedlings showed similar root length as wild-type and *brl3* mutants (S4A Fig). In addition, AtRGS1 endocytosis was not induced either with BL or BRZ (S4B Fig). FRET assays provided no evidence for a dynamic change in AtRGS1 and BRL3 interaction in *N*. *benthamiana* cells treated with 1 or 100 μM BL for 2–3 hours (S4C Fig). Therefore, we have not identified a clear role for AtRGS1 in brassinosteroid perception although GPA1 is involved in this response [36,37].

In summary, our analyses revealed the importance of the BRL3 and AtRGS1 in fine tuning growth inhibition and ROS production. Through genetic analysis, we found that BRL3 and AtRGS1 both sense glucose and flg22. They both inhibit growth in response to high glucose. They similarly function in flg22 induced ROS burst by negatively controlling ROS production. Thus, they make small adjustments in these pathways in order to achieve the optimum growth inhibition and ROS burst through dynamic interactions.

## Supporting information

S1 Fig Genotyping new mutants. **(A)** Semi quantitative PCR measurement of *brl3* transcript levels in *brl3*-1 (SALK_079612C) and *brl3*-2 (SALK_006024C). Whole plant tissue from seedlings grown in fresh 1/2 × MS liquid media for 9 days was harvested by flash freezing in liquid N2. The mRNA and cDNA were prepared with RNAeasy^TM^ (Qiagen) and Superscript III (Invitrogen), respectively, according to the manufacturer’s instructions. The PCR amplification protocol with Taq polymerase consists of an initial denaturation step at 95 °C for 5 min, followed by 30 amplification cycles at 94 °C for 30 s, 57 °C for 1 min,72 °C for 90 s and 4 °C for 1 min. Forward full-length *brl3* coding sequence primer: ATGAAACAACAATGGCAGTTCTTGA; Reverse Full length brl3 coding sequence primer: TTGTAGACATCTCCAAATCCACCTG **(B)** Genotyping of two *brl3*-2/rgs1-2 plants using the primers and protocol above. Primers used are *brl3*-2 Left genomic primer: CCAGTGAACTCGTTTGAGCTC; *brl3*-2 Right genomic primer: TTTATCGAACACTTTGTGGGC; *rgs1*-2 Left genomic primer: TGTTGATGAAAAGCCTTAGCG; *rgs1*-2 Right genomic primer: TAGCTGCTACGCTGGAGAAAC; and T-DNA left border primer: TGG TTC ACG TAG TGG GCC ATC.
(JPG)

S2 Fig Negative controls for BiFC. The negative control AtHIR2-nYFP does not complement BRL3-cYFP. Neither AtRGS1-nYFP nor GPA1-nYFP produces fluorescence by itself.
(JPG)

S3 Fig Negative control for the sugar response. **(A)** Top: 96 hr- old wt, *brl3*-2, *rgs1*-2, *brl3*-2*/rgs1*-2 mutants grown on ¼ MS media under continuous dim-light (20–40 μEinstein/m^2^/s) vertically. Bottom: 8-day-old Arabidopsis seedlings grown on ¼ MS media under continuous dim-light (20–40 μEinstein/m^2^/s) horizontally. **(B)** Root Elongation. (**C)** BRL3 and AtRGS1 are not involved in high mannitol response. (**D)** ROS burst in response to 1 μM flg22 in leaf discs (n = 16 to 24) including all the time points from 0 to 48 min.
(TIF)

S4 Fig Brassinolide (BL) assay. (**A)** *rgs1*-2 mutants show similar root growth inhibition response with BL to wt. (**B)** AtRGS1 internalization is not affected by BRZ or BL. **(C)** No change is detected in AtRGS1 and BRL3 interaction dynamics in response to BL.
(JPG)
