# Supplementary figures and images for: BRL3 and AtRGS1 cooperate to fine tune growth inhibition and ROS activation
Source: PLoS One. 2017 May 18;12(5):e0177400. doi: 10.1371/journal.pone.0177400 (PMC5436702; doi:10.1371/journal.pone.0177400)

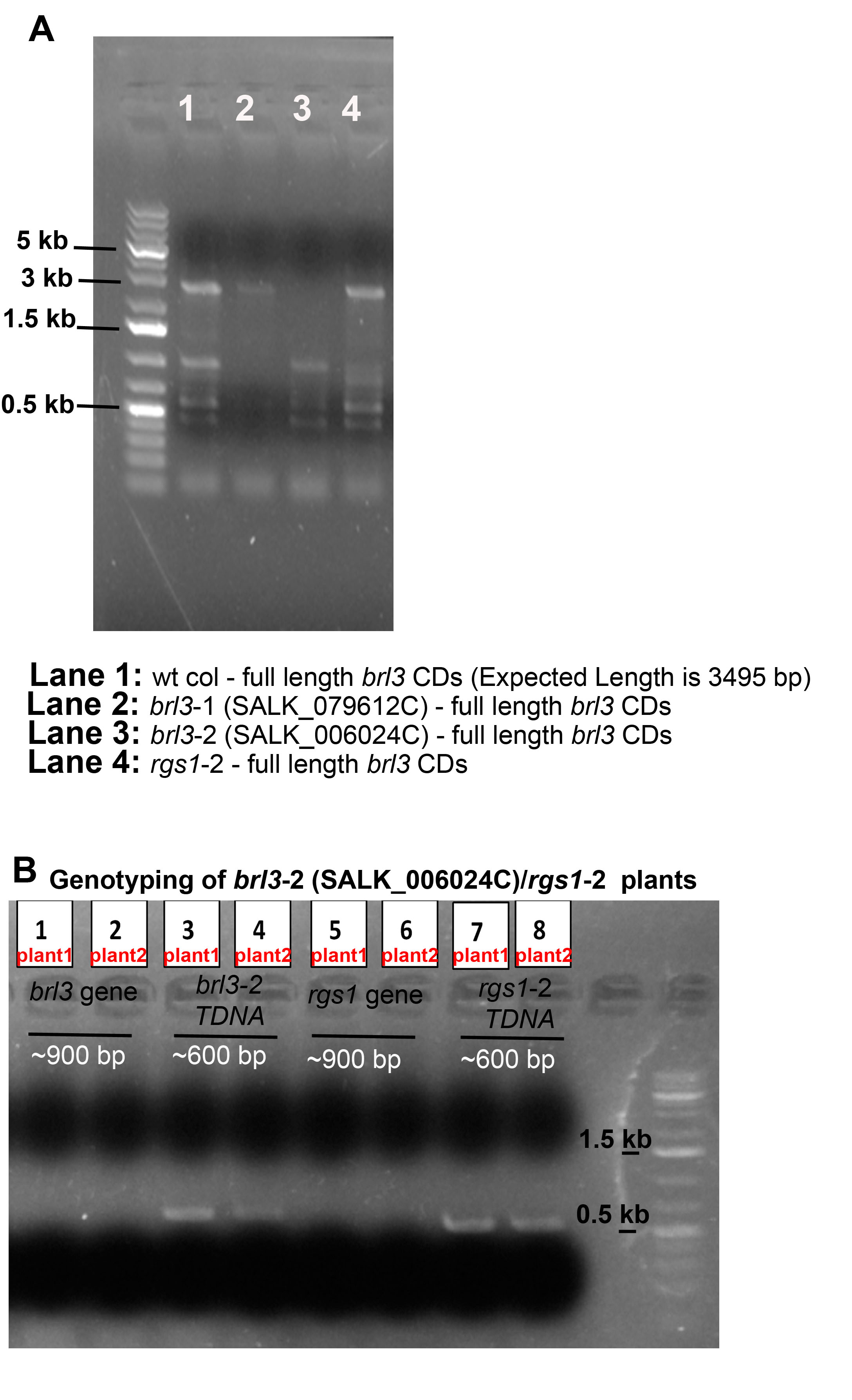

Supplement: S1 Fig — (A) Semi quantitative PCR measurement of brl3 transcript levels in brl3-1 (SALK_079612C) and brl3-2 (SALK_006024C). Whole plant tissue from seedlings grown in fresh 1/2 × MS liquid media for 9 days was harvested by flash freezing in liquid N2. The mRNA and cDNA were prepared with RNAeasyTM (Qiagen) and Superscript III (Invitrogen), respectively, according to the manufacturer’s instructions. The PCR amplification protocol with Taq polymerase consists of an initial denaturation step at 95 °C for 5 min, followed by 30 amplification cycles at 94 °C for 30 s, 57 °C for 1 min,72 °C for 90 s and 4 °C for 1 min. Forward full-length brl3 coding sequence primer: ATGAAACAACAATGGCAGTTCTTGA; Reverse Full length brl3 coding sequence primer: TTGTAGACATCTCCAAATCCACCTG (B) Genotyping of two brl3-2/rgs1-2 plants using the primers and protocol above. Primers used are brl3-2 Left genomic primer: CCAGTGAACTCGTTTGAGCTC; brl3-2 Right genomic primer: TTTATCGAACACTTTGTGGGC; rgs1-2 Left genomic primer: TGTTGATGAAAAGCCTTAGCG; rgs1-2 Right genomic primer: TAGCTGCTACGCTGGAGAAAC; and T-DNA left border primer: TGG TTC ACG TAG TGG GCC ATC. (JPG) [file pone.0177400.s001.jpg]

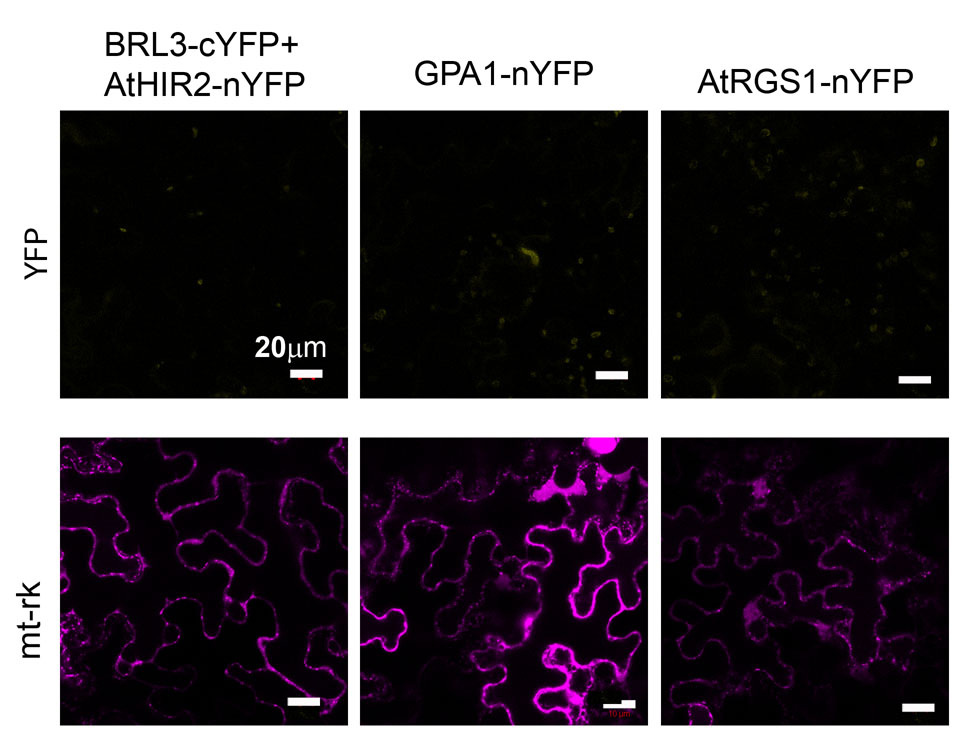

Supplement: S2 Fig — The negative control AtHIR2-nYFP does not complement BRL3-cYFP. Neither AtRGS1-nYFP nor GPA1-nYFP produces fluorescence by itself. (JPG) [file pone.0177400.s002.jpg]

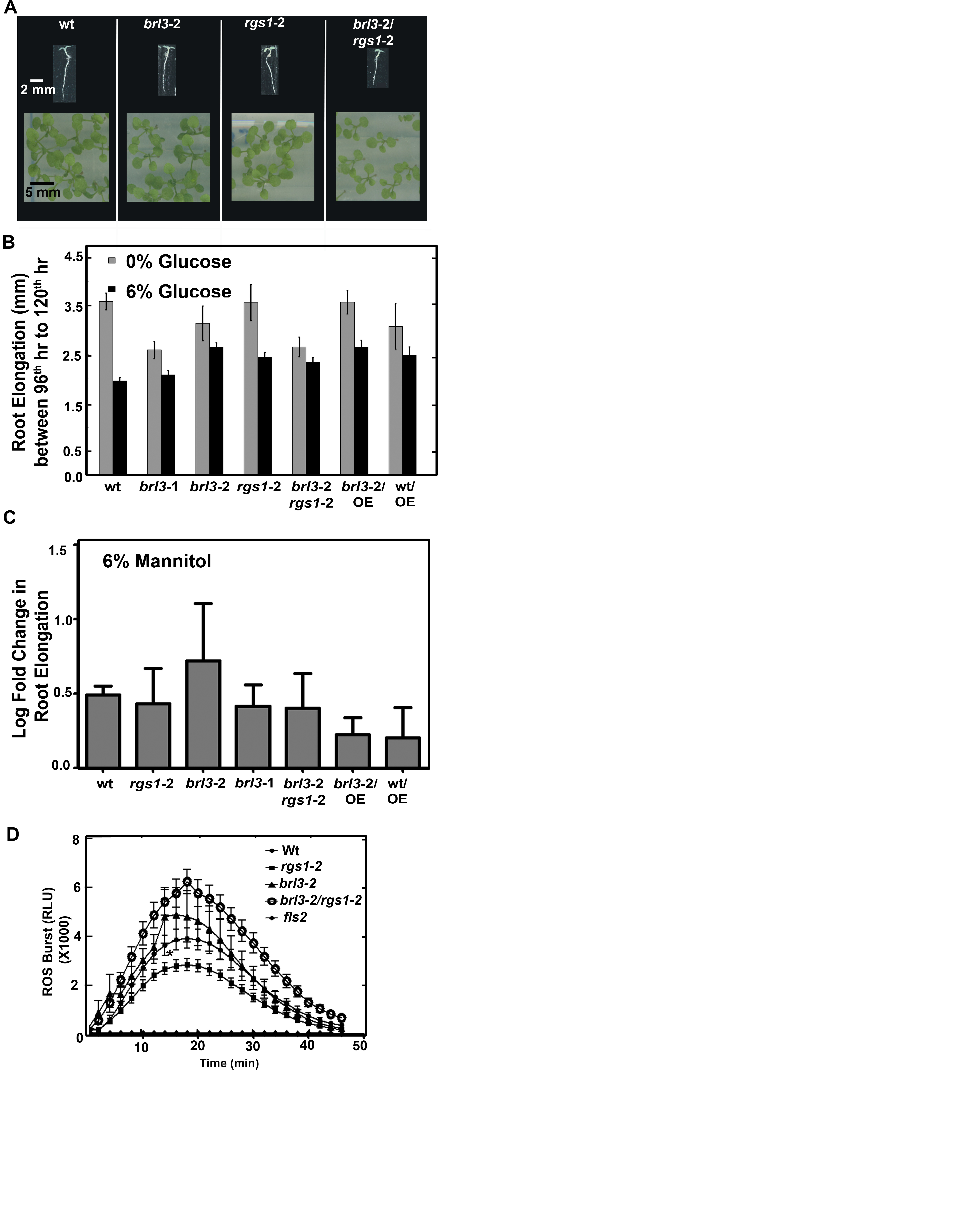

Supplement: S3 Fig — (A) Top: 96 hr- old wt, brl3-2, rgs1-2, brl3-2/rgs1-2 mutants grown on ¼ MS media under continuous dim-light (20–40 μEinstein/m2/s) vertically. Bottom: 8-day-old Arabidopsis seedlings grown on ¼ MS media under continuous dim-light (20–40 μEinstein/m2/s) horizontally. (B) Root Elongation. (C) BRL3 and AtRGS1 are not involved in high mannitol response. (D) ROS burst in response to 1 μM flg22 in leaf discs (n = 16 to 24) including all the time points from 0 to 48 min. (TIF) [file pone.0177400.s003.tif]

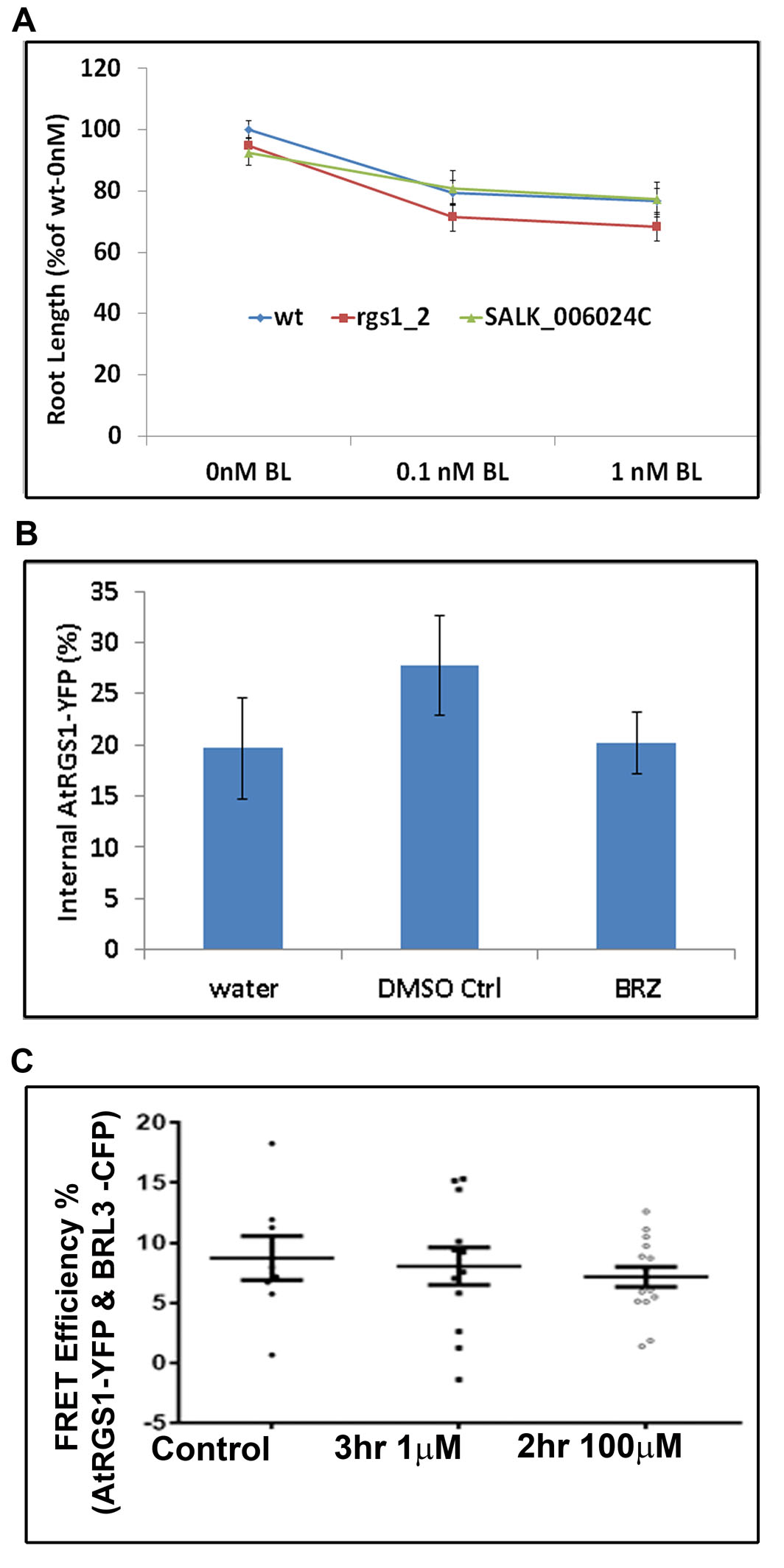

Supplement: S4 Fig — (A) rgs1-2 mutants show similar root growth inhibition response with BL to wt. (B) AtRGS1 internalization is not affected by BRZ or BL. (C) No change is detected in AtRGS1 and BRL3 interaction dynamics in response to BL. (JPG) [file pone.0177400.s004.jpg]
